# Supplementary material for: Accelerated water activation and stabilized metal-organic framework via constructing triangular active-regions for ampere-level current density hydrogen production
Source: Nat Commun. 2022 Oct 30;13:6486. doi: 10.1038/s41467-022-34278-6 (PMC9617936; doi:10.1038/s41467-022-34278-6)
Supplement: Supplementary file 1 — Supplementary Information [file 41467_2022_34278_MOESM1_ESM.pdf]

Supplementary Information

Accelerated Water Activation and Stabilized Metal-Organic Framework via Constructing Triangular Active-Regions for Ampere-Level Current Density Hydrogen Production

Fanpeng Cheng,[1] Xianyun Peng,[2] Lingzi Hu,[3] Bin Yang,[1,2] Zhongjian Li,[1,2] Chung-Li Dong,[4] Jeng-Lung Chen,[5] Liang-Ching Hsu,[5] Lecheng Lei,[1,2] Qiang Zheng,[6] Ming Qiu,[3]* Liming Dai,[7]* Yang Hou[1,2,8,9]*

[1]Key Laboratory of Biomass Chemical Engineering of Ministry of Education, College of Chemical and Biological Engineering, Zhejiang University, Hangzhou 310027, China

[2]Institute of Zhejiang University - Quzhou, Quzhou 324000, China

[3]Institute of Nanoscience and Nanotechnology, College of Physical Science and Technology, Central China Normal University, Wuhan 430079, China

[4]Department of Physics, Tamkang University, Tamsui 25137, Taiwan

[5]National Synchrotron Radiation Research Center, Hsinchu 30076, Taiwan

[6]CAS Key Laboratory of Standardization and Measurement for Nanotechnology, CAS Center for Excellence in Nanoscience, National Center for Nanoscience and Technology, Beijing, 100190 China

[7]Australian Carbon Materials Centre (A-CMC), School of Chemical Engineering, University of New South Wales, Sydney, NSW 2052, Australia

[8]School of Biological and Chemical Engineering, NingboTech University, Ningbo 315100, China

[9]Donghai Laboratory, Zhoushan, China

*Corresponding author. Email: qium@mail.ccnu.edu.cn; l.dai@unsw.edu.au; yhou@zju.edu.cn

Experimental section

Materials: 1,4-dicarboxybenzene acid (BDC, ≥99%) and cobalt (II) nitrate hexahydrate ($Co(NO_3)_2·6H_2O$, ≥99%) were purchased from Macklin Biochemical Co., Ltd; 1,4-benzenedimethanethiol (BDMT, ≥95%) was obtained from Bide Pharmatech Ltd; Nickel (II) chloride hexahydrate ($NiCl_2·6H_2O$, ≥98.0%), iron (III) chloride hexahydrate ($FeCl_3·6H_2O$, ≥99.0%), potassium hydroxide (KOH, ≥85.0%), zinc (II) nitrate hexahydrate ($Zn(NO_3)_2·6H_2O$, ≥99.0%), N, N-dimethylformamide (DMF, ≥99.5%) and ethanol absolute (≥99.7%) were bought from Sinopharm Chemical Reagent Co., Ltd. The water used was deionized (DI) water. All the chemicals were used as obtained without further purification.

Synthesis of 0.32 wt.% S-NiBDC. First, 119 mg of $NiCl_2·6H_2O$, 83 mg of BDC, and 0.85 mg of BDMT were dissolved in a mixed solvent of water, ethanol, and DMF (volume ratio = 1:1:16 mL), and stirred for 20 min to form a homogeneous solution. The obtained solution was transferred into a Teflon-lined autoclave with a piece of NF (1 cm × 3 cm) and then heated at 150 °C for 3 h in an oven, followed by naturally cooling down to room temperature. After being repeatedly washed with DMF, ethanol, and DI water, and dried in an oven at 60 °C, the final product of 0.32 wt.% S-NiBDC grown on NF was obtained, which has an area-specific S-NiBDC mass density of about 1.3 mg cm^{-2}.

Synthesis of 0.65 wt.% S-NiBDC. First, 119 mg of $NiCl_2·6H_2O$, 83 mg of BDC, and 2.6 mg of BDMT were dissolved in a mixed solvent of water, ethanol, and DMF (volume ratio = 1:1:16 mL), and stirred for 20 min to form a homogeneous solution. The obtained solution was transferred into a Teflon-lined autoclave with a piece of NF (1 cm × 3 cm) and then heated at 150 °C for 3 h in an oven, followed by naturally cooled down to room temperature. After being repeatedly washed with DMF, ethanol, and DI water, and dried in an oven at 60 °C, the final product of 0.65 wt.% S-NiBDC grown on NF was obtained, which has an area-specific S-NiBDC mass density of about 1.3 mg cm^{-2}.

Synthesis of 2.12 wt.% S-NiBDC. First, 119 mg of $NiCl_2·6H_2O$, 83 mg of BDC, and 6.0 mg of BDMT were dissolved in a mixed solvent of water, ethanol, and DMF

(volume ratio = 1:1:16 mL), and stirred for 20 min to form a homogeneous solution. The obtained solution was transferred into a Teflon-lined autoclave with a piece of NF (1 cm × 3 cm) and then heated at 150 °C for 3 h in an oven, followed by naturally cooled down to room temperature. After being repeatedly washed with DMF, ethanol, and DI water, and dried in an oven at 60 °C, the final product of 2.12 wt.% S-NiBDC grown on NF was obtained, which has an area-specific S-NiBDC mass density of about 1.1 mg cm^{-2}.

Synthesis of S-CoBDC. First, 145.5 mg of $Co(NO_3)_2·6H_2O$, 83 mg of BDC, and 4.3 mg of BDMT were dissolved in a mixed solvent of water, ethanol, and DMF (volume ratio = 1:1:16 mL), and stirred for 20 min to form a homogeneous solution. The obtained solution was transferred into a Teflon-lined autoclave with a piece of NF (1 cm × 3 cm) and then heated at 150 °C for 3 h in an oven, followed by naturally cooled down to room temperature. After being repeatedly washed with DMF, ethanol, and DI water, and dried in an oven at 60 °C, the final product of S-CoBDC grown on NF was obtained. The loading amount of the S-CoBDC on NF was determined to be about 1.2 mg cm^{-2}.

Synthesis of CoBDC. First, 145.5 mg of $Co(NO_3)_2·6H_2O$ and 83 mg of BDC were dissolved in a mixed solvent of water, ethanol, and DMF (volume ratio = 1:1:16 mL), and stirred for 20 min to form a homogeneous solution. The obtained solution was transferred into a Teflon-lined autoclave with a piece of NF (1 cm × 3 cm) and then heated at 150 °C for 3 h in an oven, followed by naturally cooled down to room temperature. After being repeatedly washed with DMF, ethanol, and DI water, and dried in an oven at 60 °C, the final product of CoBDC grown on NF was obtained. The loading amount of the CoBDC on NF was determined to be about 1.4 mg cm^{-2}.

Synthesis of S-FeBDC. First, 135 mg of $FeCl_3·6H_2O$, 83 mg of BDC, and 4.3 mg BDMT were dissolved in a mixed solvent of water, ethanol, and DMF (volume ratio = 1:1:16 mL), and stirred for 20 min to form a homogeneous solution. The obtained solution was transferred into a Teflon-lined autoclave with a piece of NF (1 cm × 3 cm) and then heated at 150 °C for 3 h in an oven, followed by naturally cooled down to room temperature. After being repeatedly washed with DMF, ethanol, and DI water,

and dried in an oven at 60 °C, the final product of S-FeBDC grown on NF was obtained. The loading amount of the S-FeBDC on NF was determined to be about 1.5 mg cm^{-2}.

Synthesis of FeBDC. First, 135 mg of $FeCl_3·6H_2O$ and 83 mg of BDC were dissolved in a mixed solvent of water, ethanol, and DMF (volume ratio = 1:1:16 mL), and stirred for 20 min to form a homogeneous solution. The obtained solution was transferred into a Teflon-lined autoclave with a piece of NF (1 cm × 3 cm) and then heated at 150 °C for 3 h in an oven, followed by naturally cooled down to room temperature. After being repeatedly washed with DMF, ethanol, and DI water, and dried in an oven at 60 °C, the final product of FeBDC grown on NF was obtained. The loading amount of the FeBDC on NF was determined to be about 1.6 mg cm^{-2}.

Characterization: The morphologies of the as-prepared catalysts were examined by FESEM (Hitachi SU-8010) with EDX (Oxford, X-max80), TEM (HT7700), and HRTEM (Tecnai G2 F20 S-TWIN). The crystal structures of the as-prepared catalysts were measured by XRD (ZETIUM DY powder XRD unit) using Cu Kα radiation at 4.0 KW. The chemical environments of as-prepared catalysts were measured by XPS (Escalab 250Xi) with Al Kα radiation. Raman spectra of the as-prepared catalysts were measured with a HORIBA/XploRA PLUS at 532 nm. FT-IR spectra were recorded on ThermoFisher Nicolet 6700 at room temperature. The contents of nickel and sulfur in the as-prepared catalysts were analyzed by ICP-AES (Agilent 720). The N_2 adsorption-desorption curves of the as-prepared catalysts were tested by BET (Micromeritics/3FLEX-3MP). The water-droplet contact angles were measured by SDC-100S.

Calculation method: The reaction pathways for H_2 in HER are simulated as the following:

$$* + H_2O \rightarrow H_2O* \quad \text{S1}$$

$$H_2O* + e^- \rightarrow H* + OH^- \quad \text{S2}$$

$$H* + (H_2O + e^-) \rightarrow H_2* + OH^- \quad \text{S3}$$

$$H_2* \rightarrow * + H_2\uparrow \quad \text{S4}$$

In these equations, the * denotes the catalytic active site on the surface; H_2O*, H*,

and H_2* denote the reaction intermediates with adsorbed groups of H_2O, H, and H_2, respectively.

For the free energies calculations, the zero-point energy (ZPE) and entropy corrections are taken into account in the absorption energies parts. The equation is as follows:

$$\Delta G_{ads} = \Delta E_{\mathrm{ads}} + \Delta ZPE - T\Delta S \qquad \text{S5}$$

For each elementary reaction step, the reaction free energy is calculated by:

$$\Delta G = \Delta E + \Delta ZPE - T\Delta S + \Delta G_{\mathrm{U}} + \Delta G_{\mathrm{pH}} \qquad \text{S6}$$

The reaction energy difference between reactant and product is denoted as ΔE; the free energy of product ΔG_U is dependent on the electrode potential V and electron charge e.

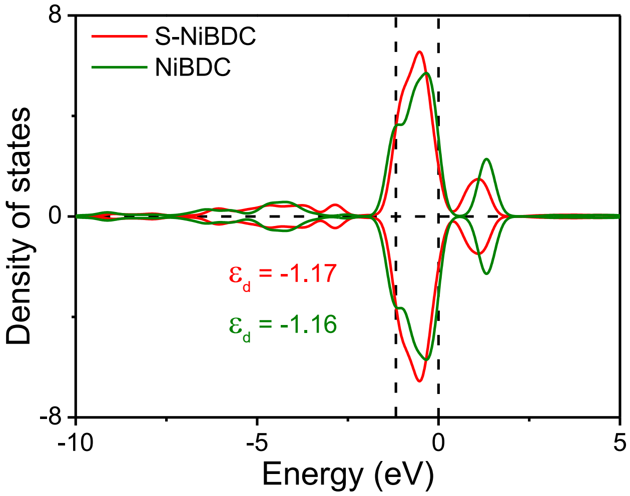

Figure S1. PDOS of *d*-states for NiBDC and S-NiBDC.

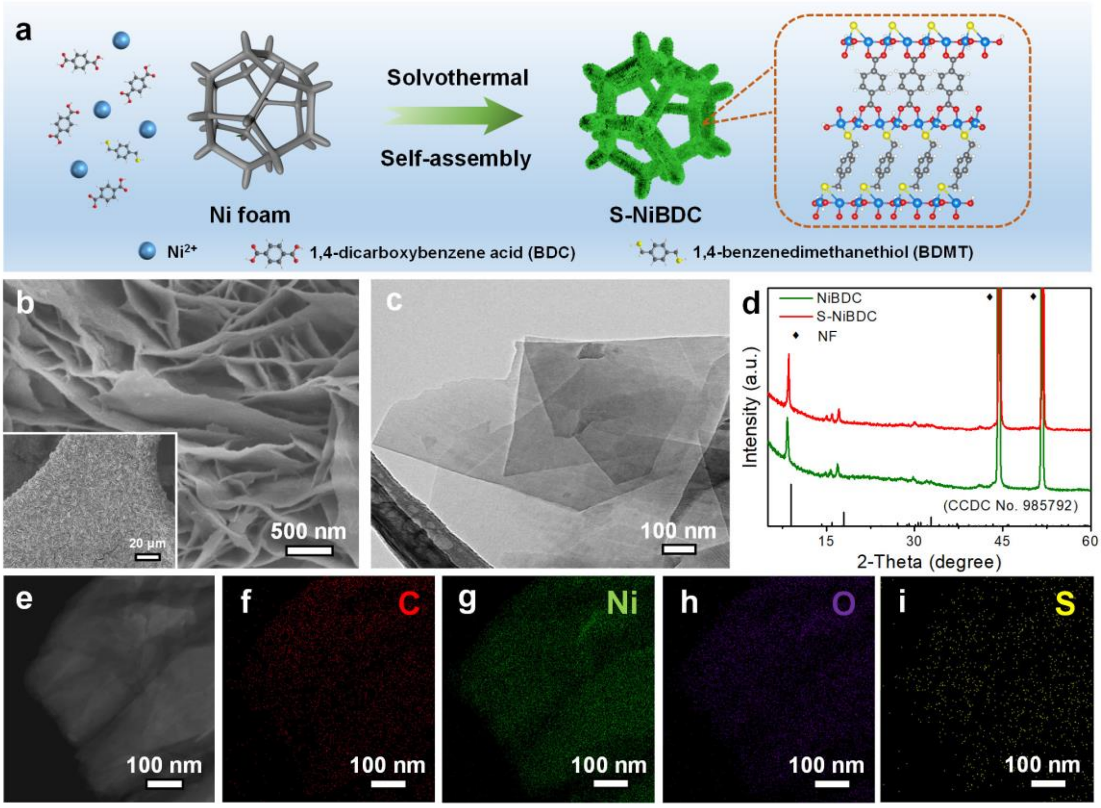

Figure S2. (a) Schematic of the synthesis process of S-NiBDC. (b-c) FESEM and TEM images of S-NiBDC. Inset: low-magnification FESEM image. (d) XRD patterns of S-NiBDC and NiBDC. (e-i) HAADF-STEM image and EDX elemental mapping images of S-NiBDC.

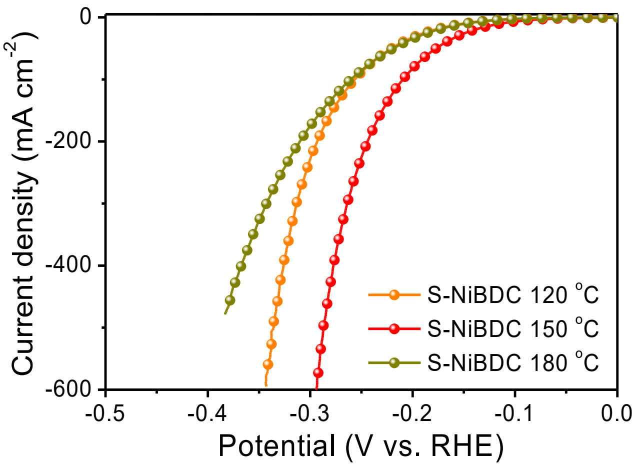

Figure S3. Polarization curves of S-NiBDC synthesized under different solvothermal temperatures of 120 °C, 150 °C, and 180 °C. The S-NiBDC catalyst prepared at 150 °C displayed the highest HER activity.

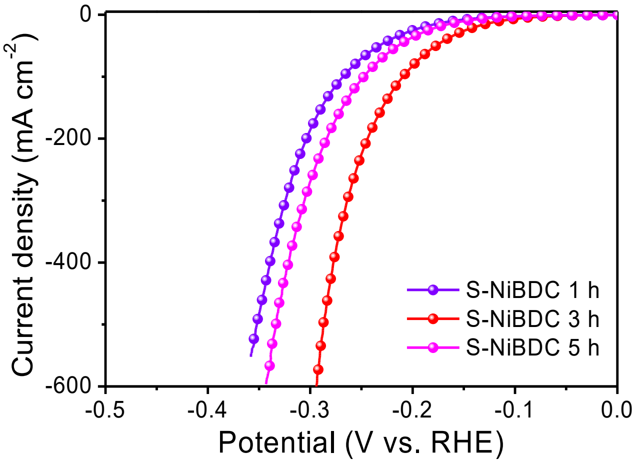

Figure S4. Polarization curves of S-NiBDC synthesized under different solvothermal times of 1 h, 3 h, and 5 h at 150 ºC. The S-NiBDC catalyst prepared at 150 ºC for 3 h displayed the highest HER activity.

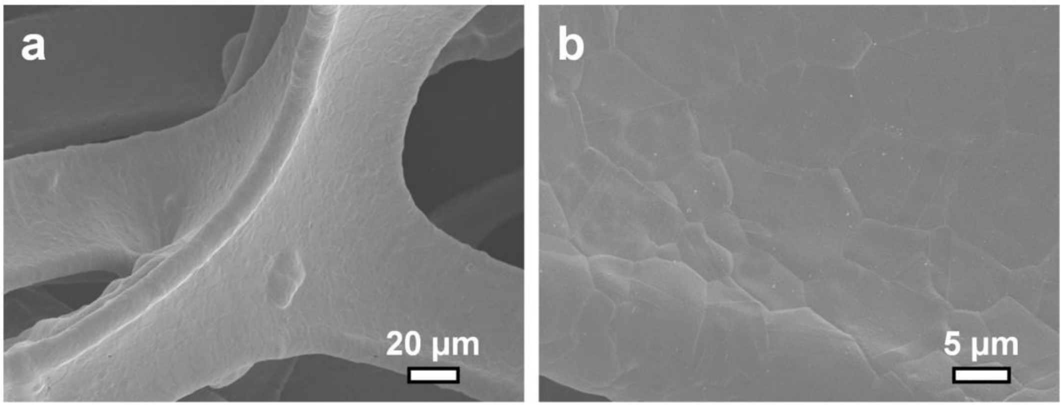

Figure S5. (a-b) FESEM images of NF.

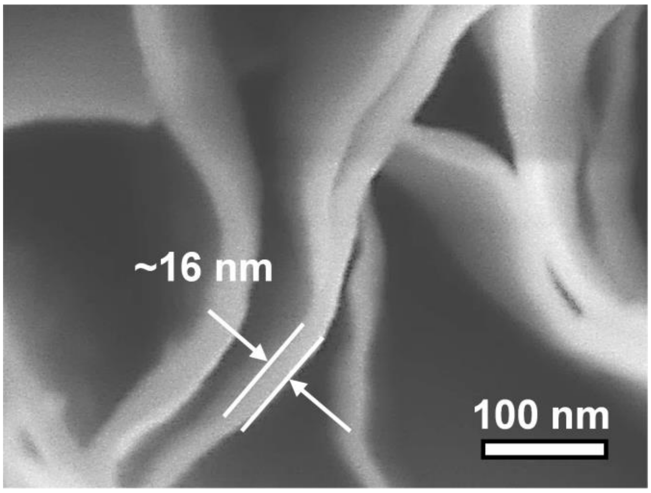

Figure S6. FESEM image of S-NiBDC.

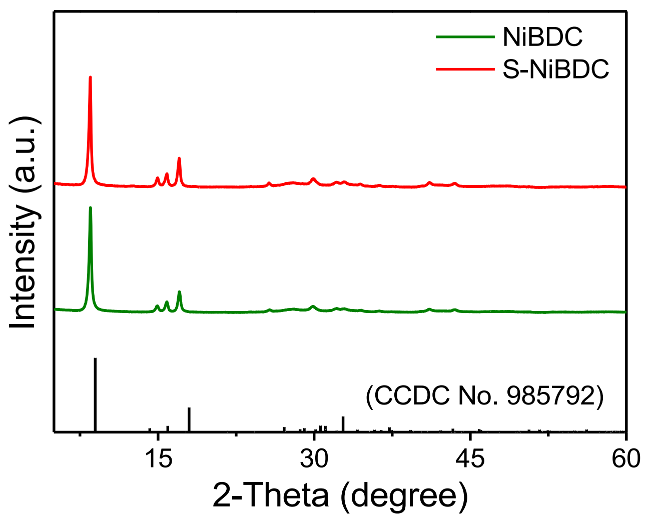

Figure S7. XRD patterns of S-NiBDC and NiBDC without substrates.

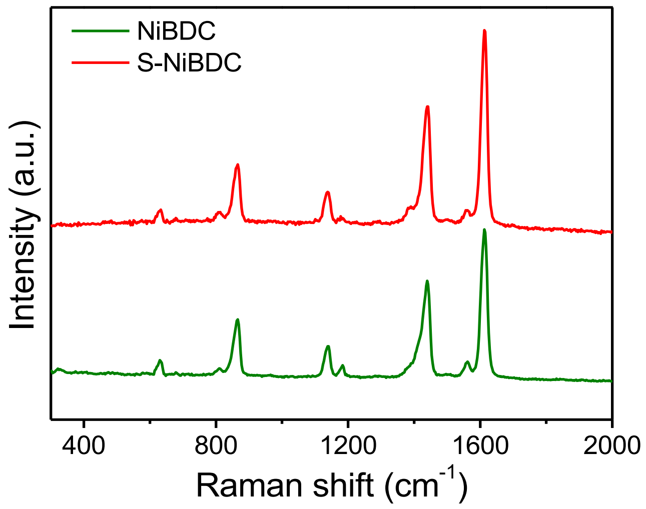

Figure S8. Raman spectra of S-NiBDC and NiBDC.

Raman spectra of NiBDC and S-NiBDC share similar bands at 1618 and 1444 cm^{-1}, which are attributed to the in- and out-of-phase stretching modes of the coordinated carboxylate groups bond with metal centers. The C-H stretching region of the benzene ring is set at 865, 808, and 623 cm^{-1}.

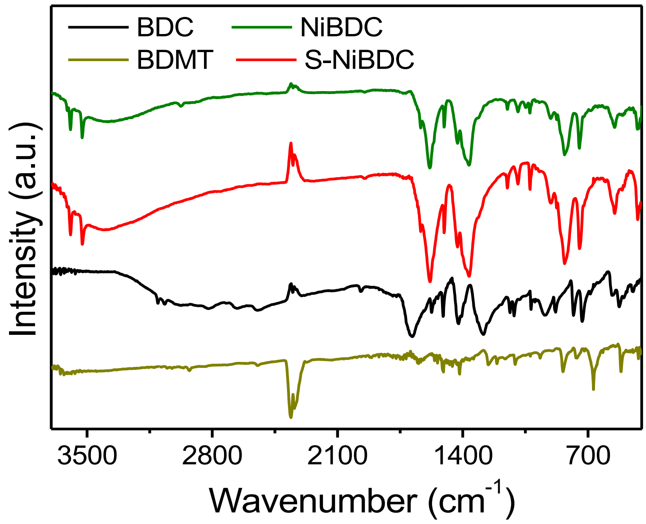

Figure S9. FT-IR spectra of BDC, BDMT, NiBDC, and S-NiBDC.

FT-IR spectrum of S-NiBDC presents adsorption peaks at 1576 and 1364 cm^{-1} that verified the existence of carboxylate groups.

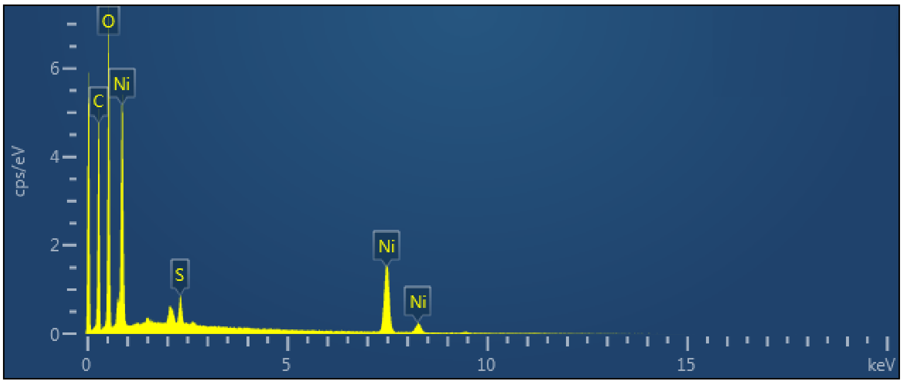

Figure S10. EDX elemental spectrum of S-NiBDC.

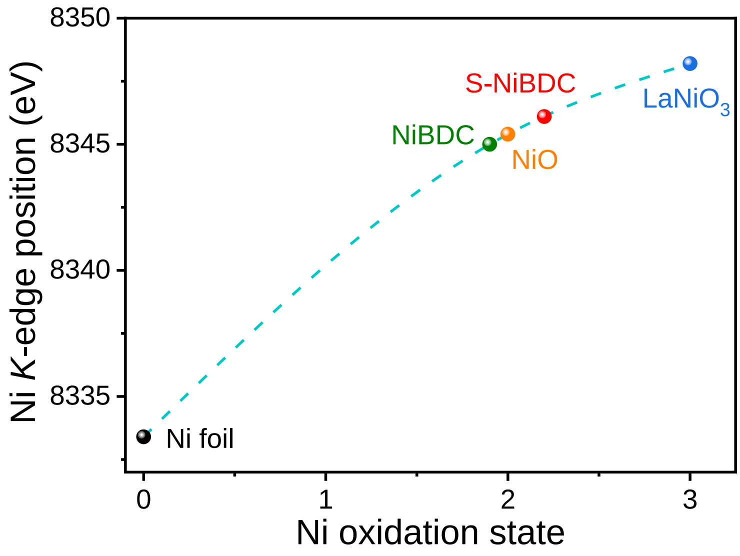

Figure S11. The oxidation states of Ni species plotted against Ni *K*-edge position obtained from the first derivative method.

The Ni foil and NiO references are taken from the standard testing. The chemical value for $LaNiO_3$ (III) standard reference is taken from the literature (J. Mater. Chem., 2011, 21, 18592-18596). The Ni *K*-edge energy position correlates nonlinearly with the formal valences of the reference samples.

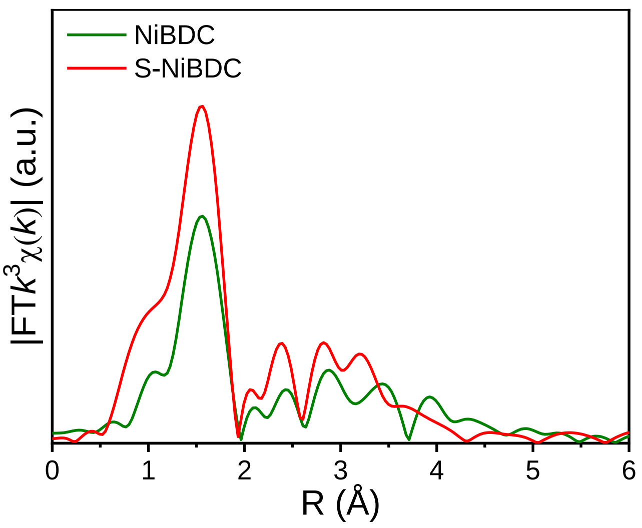

Figure S12. EXAFS spectra of S-NiBDC and NiBDC.

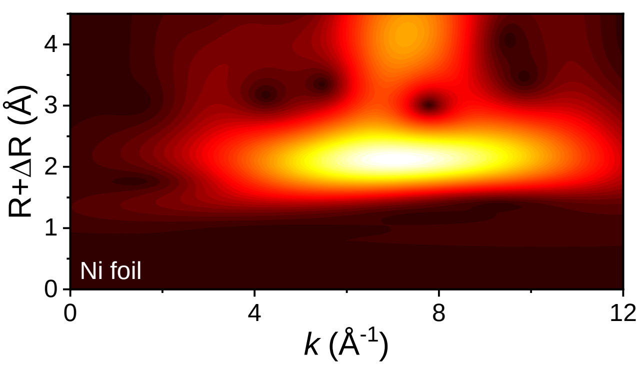

Figure S13. WT-EXAFS spectrum of Ni foil.

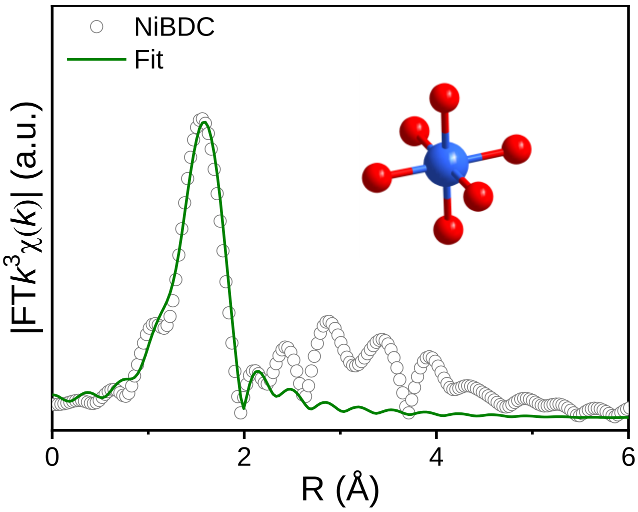

Figure S14. Fourier transform-EXAFS fitting results of NiBDC.

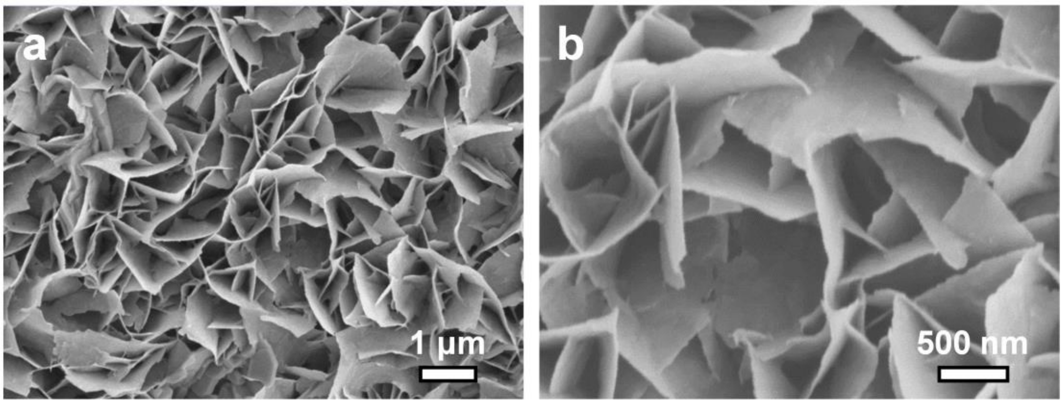

Figure S15. (a-b) FESEM images of NiBDC.

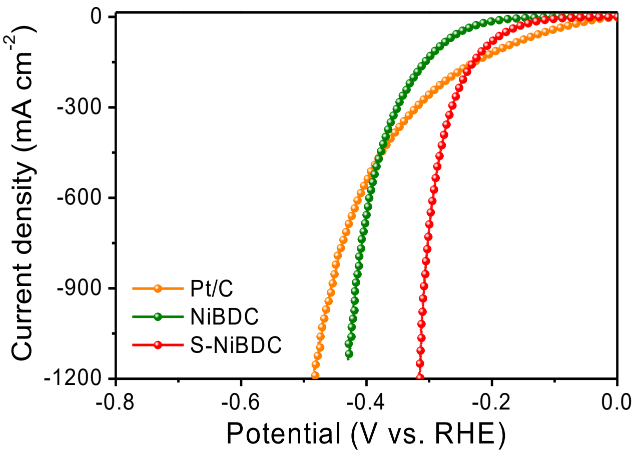

Figure S16. Polarization curves of NiBDC, S-NiBDC, and Pt/C.

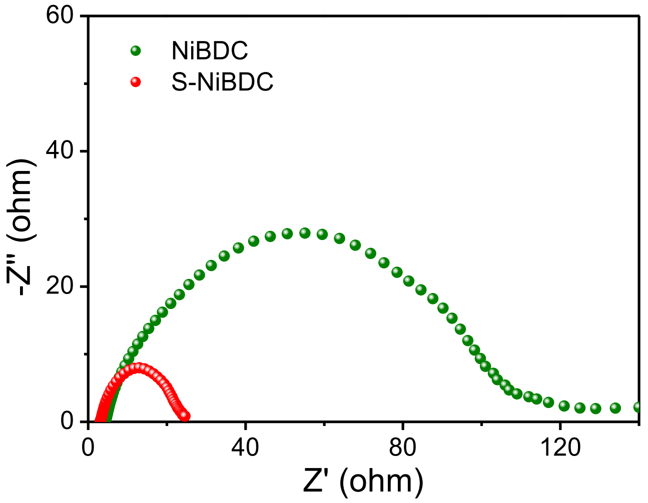

Figure S17. EIS Nyquist plots of S-NiBDC and NiBDC.

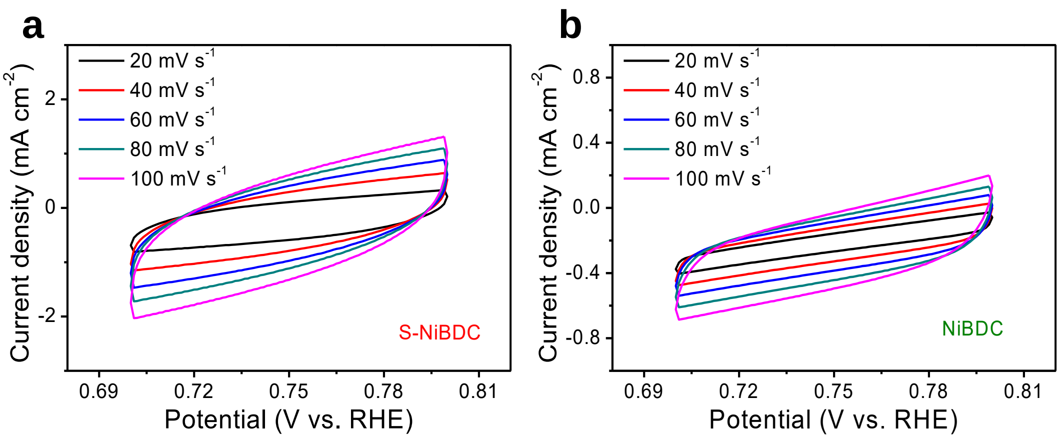

Figure S18. (a-b) CV curves of S-NiBDC and NiBDC at different scan rates in 1.0 M KOH.

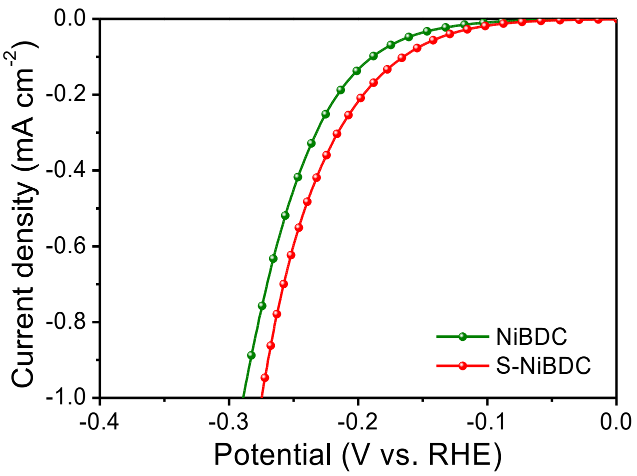

Figure S19. Polarization curves of NiBDC and S-NiBDC normalized by ECSA.

The ECSA of each sample can be evaluated from C_{dl} according to the following equation:

$$ECSA=C_{dl}/C_s$$

where C_s is the specific capacitance of the sample or the capacitance of an atomically smooth planar surface of the material per unit area under identical electrolyte conditions. The C_s is usually found to be in the range of 0.02-0.06 mF cm^{-2}, and it is assumed as 0.04 mF cm^{-2} in the calculations of ECSA.

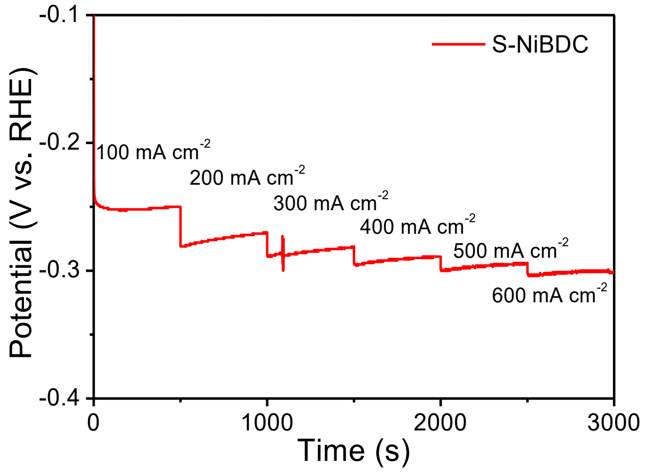

Figure S20. Multi-step chronopotentiometric curve of S-NiBDC.

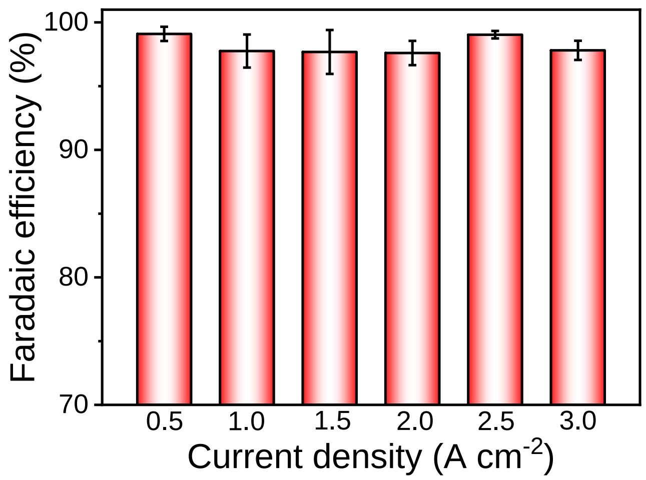

Figure S21. Faradaic efficiency tests for S-NiBDC at different current densities. Error bars correspond to the standard deviation of the three measurements.

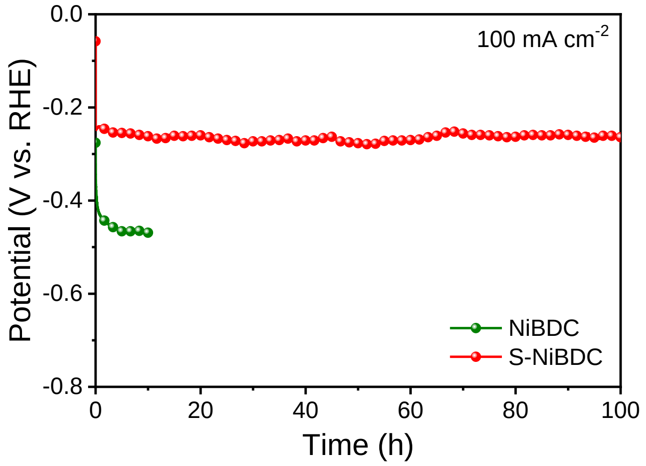

Figure S22. Chronopotentiometric curves at 100 mA cm^{-2} for NiBDC and S-NiBDC.

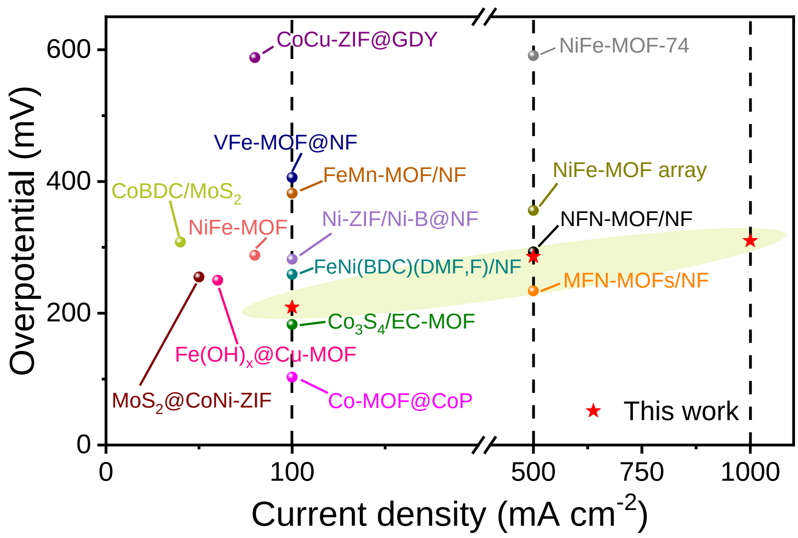

Figure S23. Comparison of HER performances of S-NiBDC and other reported MOF-based HER catalysts at different current densities.

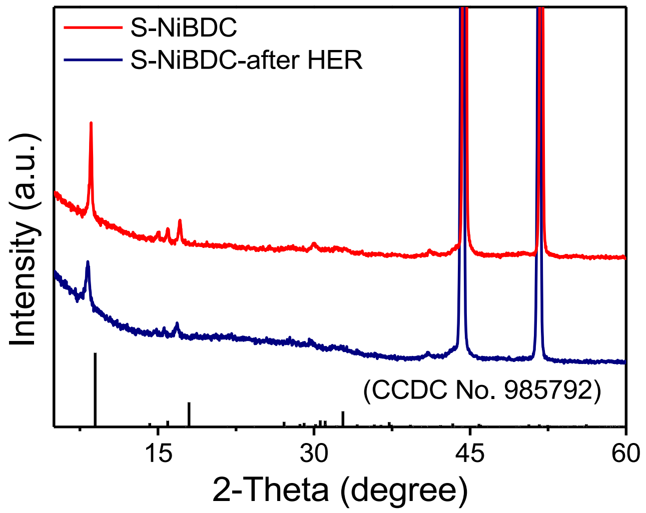

Figure S24. XRD patterns of S-NiBDC before and after HER tests.

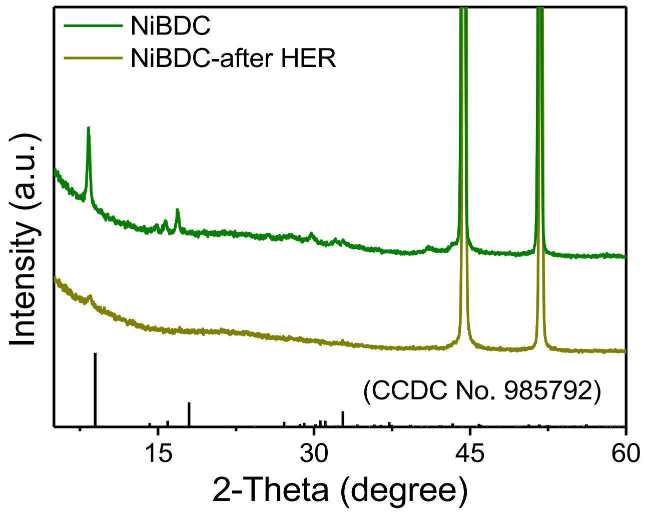

Figure S25. XRD patterns of NiBDC before and after HER tests.

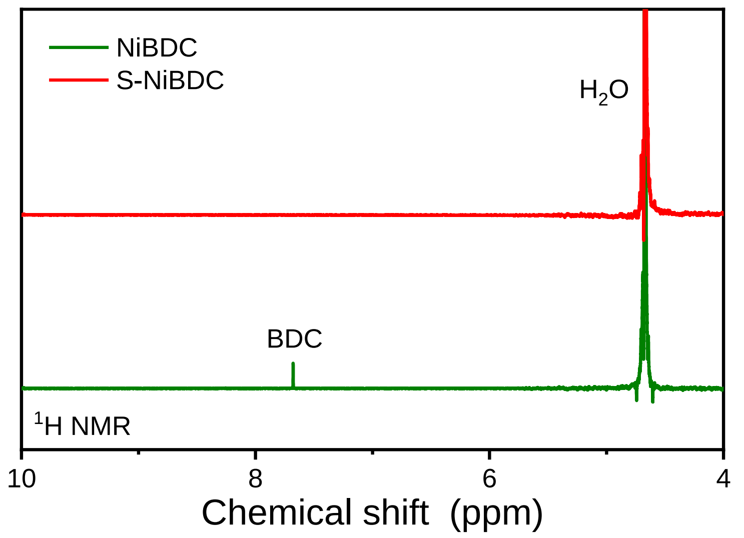

Figure S26. ^{1}H-NMR spectra of the electrolytes after HER tests for NiBDC and S-NiBDC.

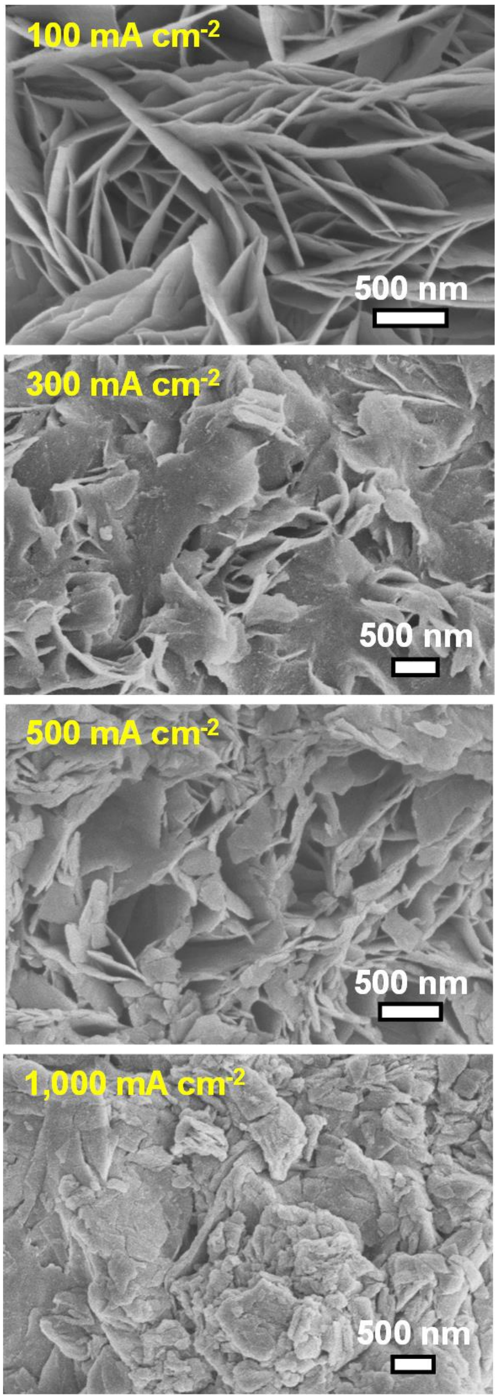

Figure S27. FESEM images of NiBDC after HER tests at different current densities.

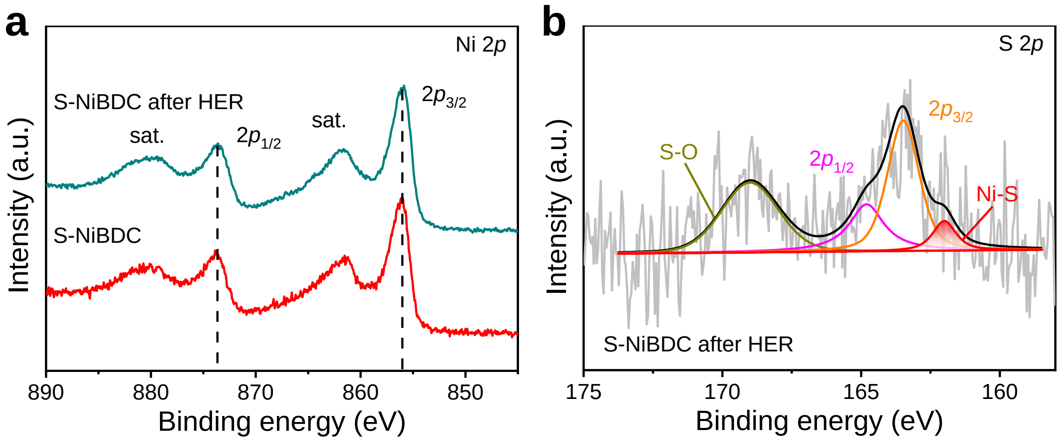

Figure S28. High-resolution XPS spectra for (a) Ni 2*p* and (b) S 2*p* of S-NiBDC after HER tests.

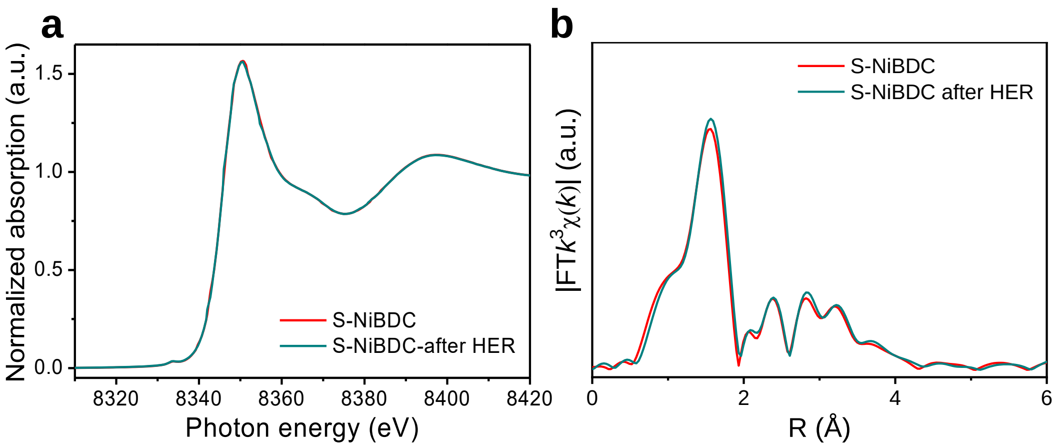

Figure S29. (a) Ni *K*-edge XANES and (d) k^3-weighted EXAFS spectra of S-NiBDC before and after HER tests.

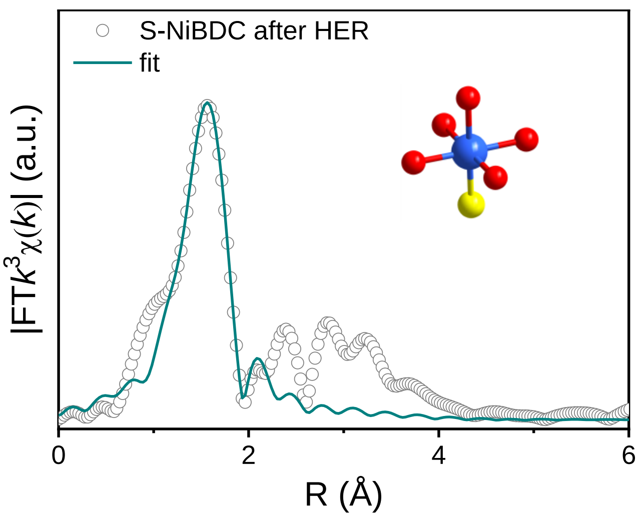

Figure S30. Fourier transform-EXAFS fitting results of S-NiBDC after HER tests.

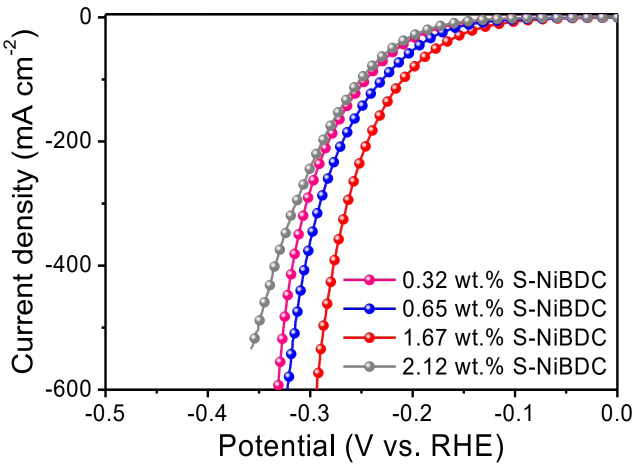

Figure S31. Polarization curves of S-NiBDC samples with different S doping amounts.

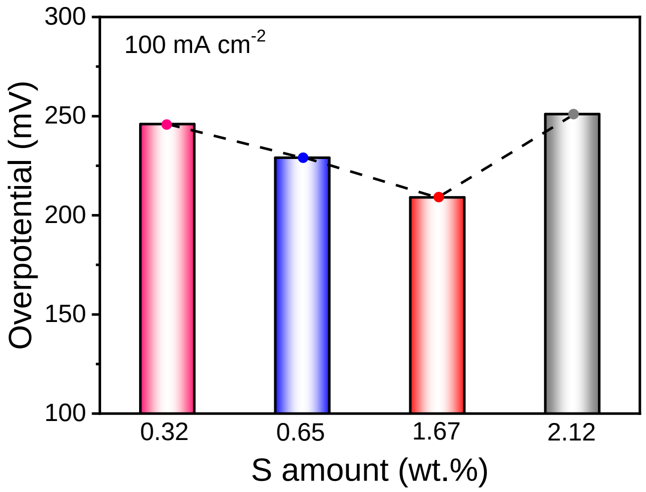

Figure S32. Overpotentials at 100 mA cm^{-2} for S-NiBDC samples with different S doping amounts.

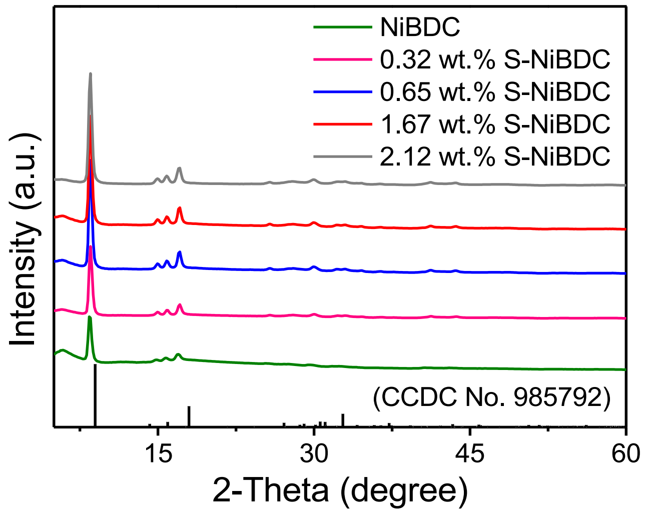

Figure S33. XRD patterns of NiBDC and S-NiBDC samples with different S doping amounts.

The XRD patterns for S-NiBDC samples with different S doping amounts exhibit identical peaks, revealing the existence of MOFs structure.

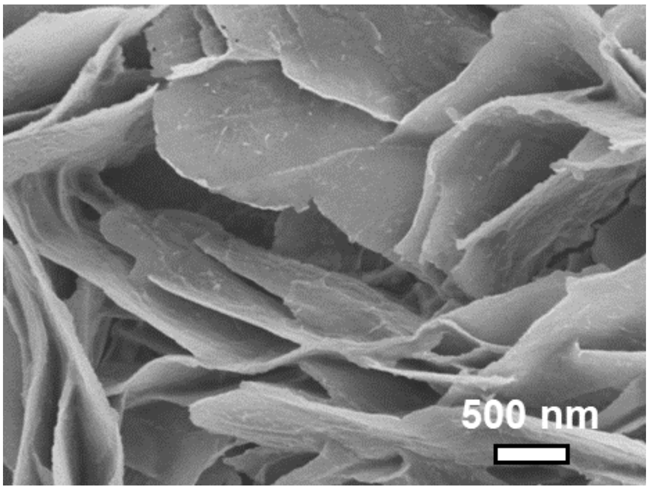

Figure S34. FESEM image of 0.32 wt.% S-NiBDC.

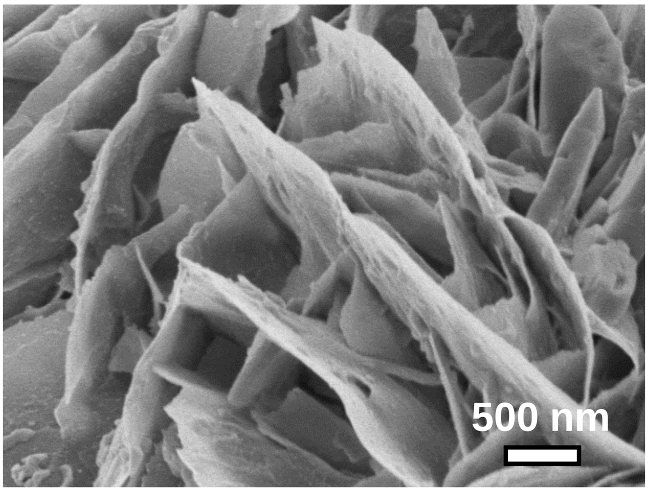

Figure S35. FESEM image of 0.65 wt.% S-NiBDC.

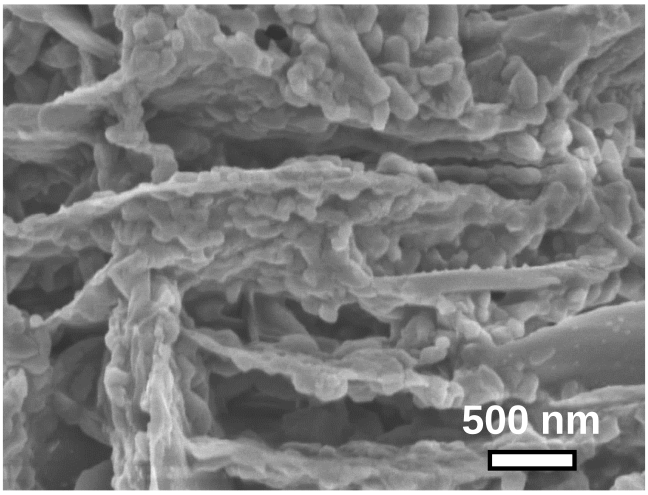

Figure S36. FESEM image of 2.12 wt.% S-NiBDC.

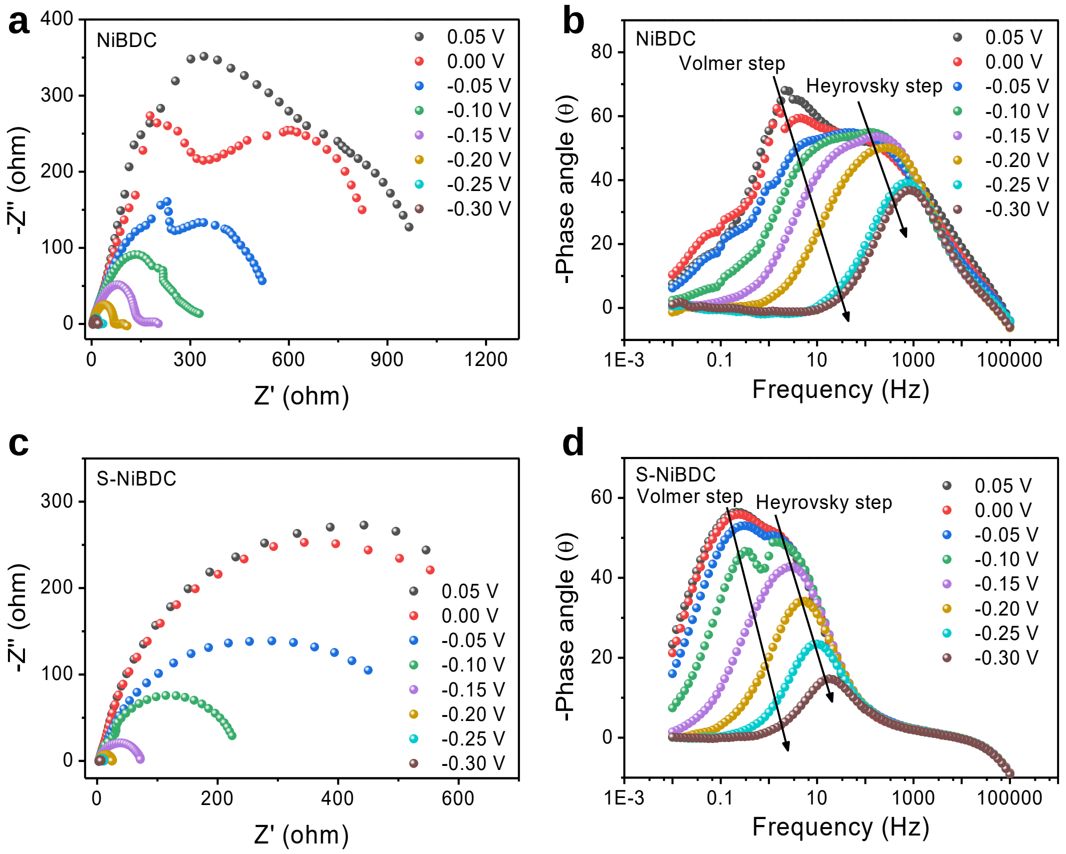

Figure S37. Operando Nyquist and Bode plots for NiBDC (a-b) and S-NiBDC (c-d) in 1.0 M KOH.

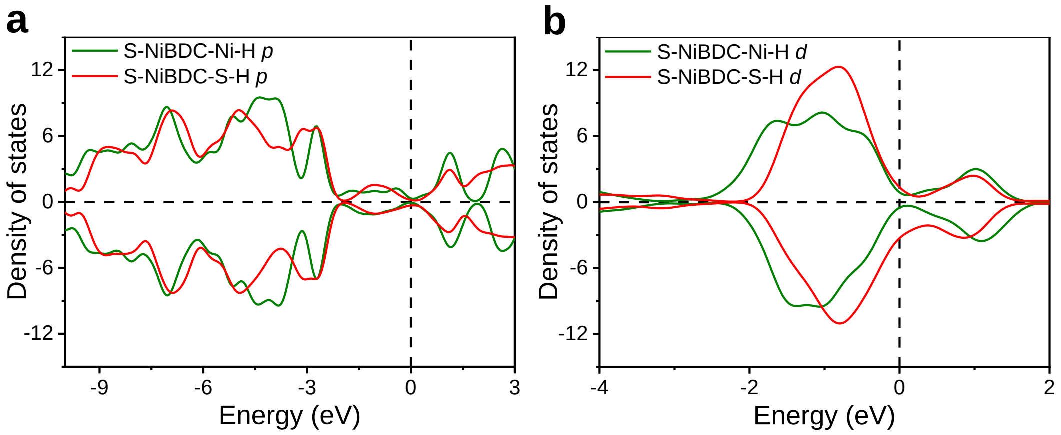

Figure S38. PDOS of *p*- and *d*- states of different sites for S-NiBDC with H binding.

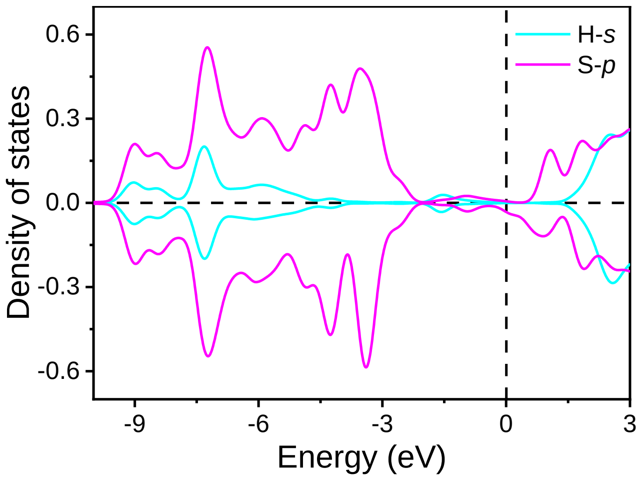

Figure S39. PDOS of H and S atoms.

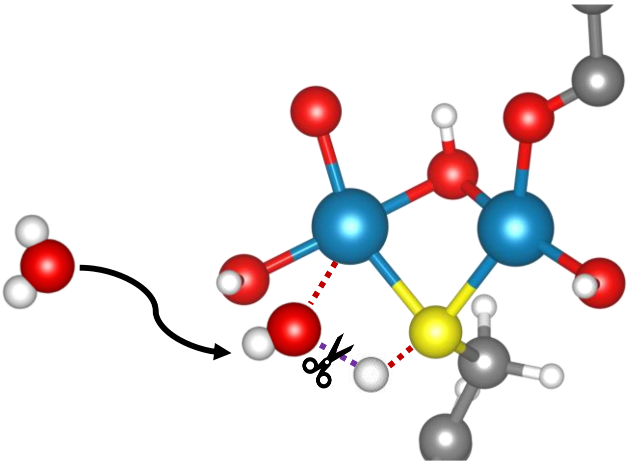

Figure S40. Schematic illustration of water activation for S-NiBDC in alkaline solution.

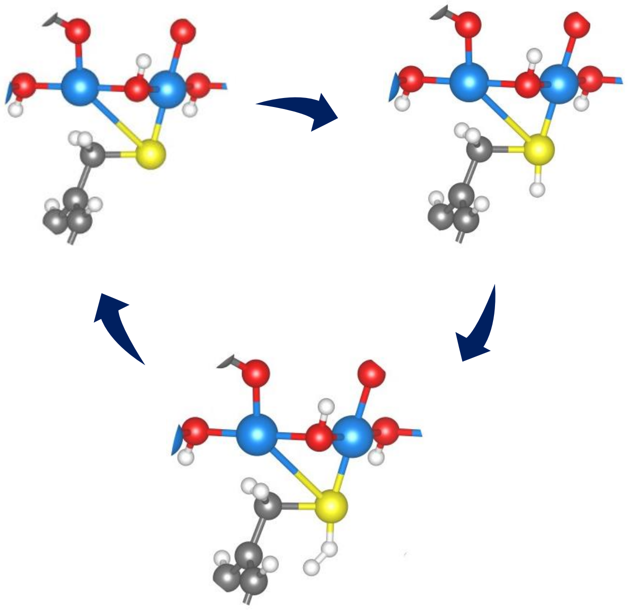

Figure S41. Schematic illustration of the HER process for S-NiBDC in alkaline solution.

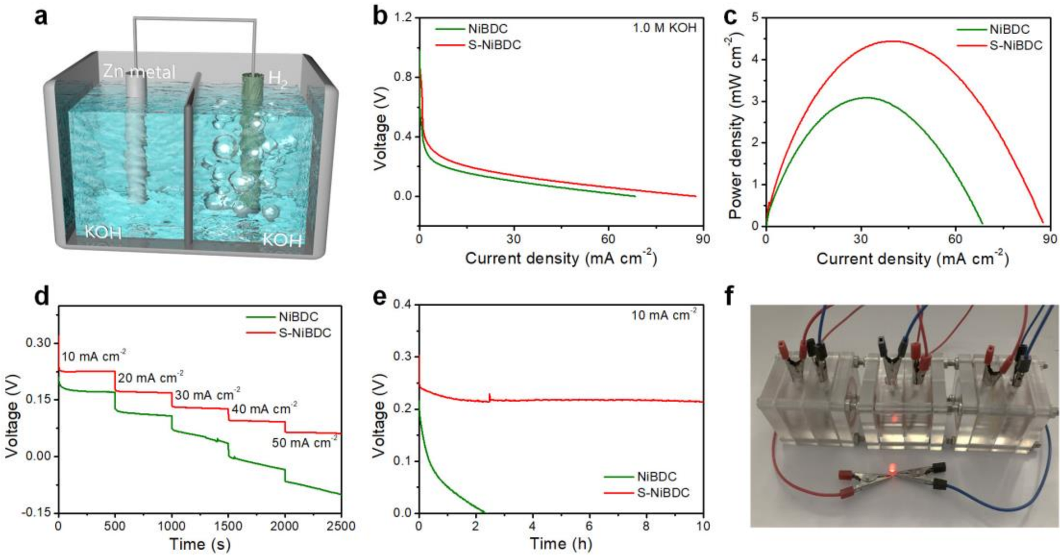

Figure S42. (a) Schematic illustration of the alkaline Zn-H_2O cell. (b) Charge polarization curves of S-NiBDC and NiBDC. (c) Corresponding power density curves. (d) Chronopotentiometric response of the Zn-H_2O cell using S-NiBDC and NiBDC at current densities ranging from 10 to 50 mA cm^{-2} during the discharging process. (e) Long-term durability tests for Zn-H_2O cells using S-NiBDC and NiBDC. (f) Digital photograph of a red light-emitting diode lighted by three S-NiBDC-based Zn-H_2O cells connected in series.

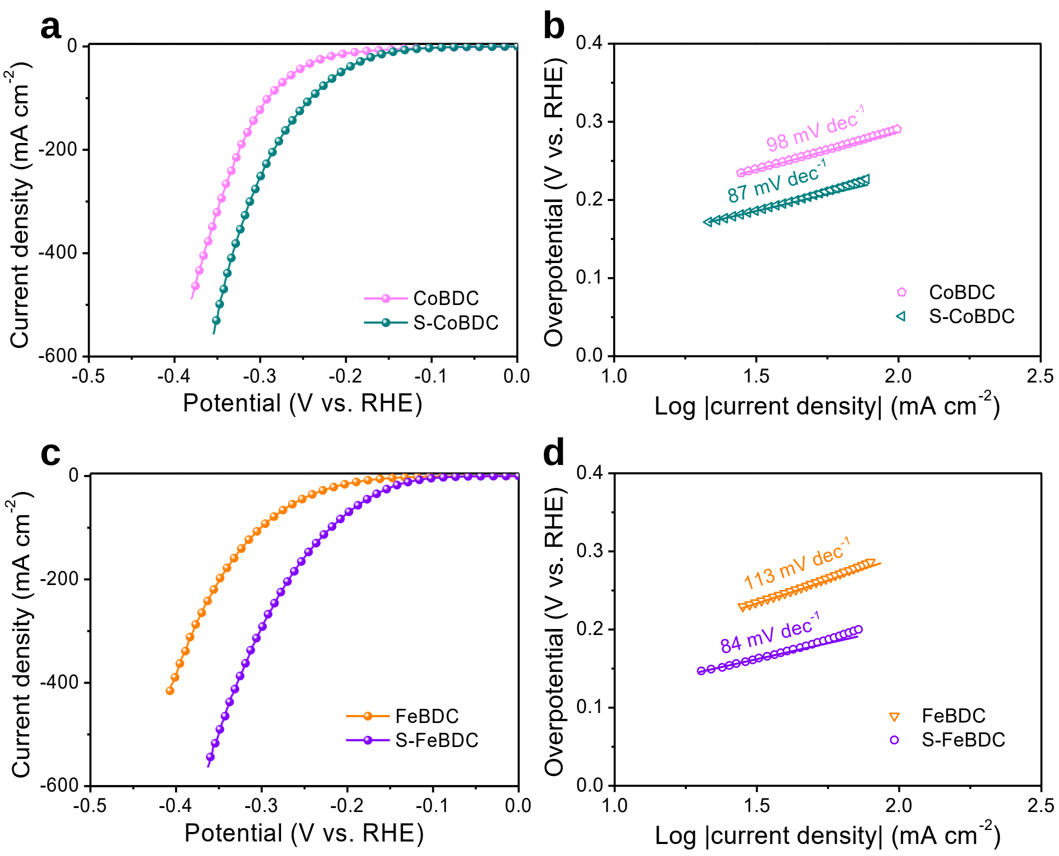

Figure S43. (a) Polarization curves and (b) Tafel slopes of S-CoBDC and CoBDC. (c) Polarization curves and (d) Tafel slopes of S-FeBDC and FeBDC.

The required overpotentials to reach 100 mA cm^{-2} are 241 and 218 mV for S-CoBDC and S-FeBDC, respectively, lower than that of CoBDC (292 mV) and FeBDC (301 mV) without adding S species. The corresponding Tafel slopes for S-CoBDC and S-FeBDC are 87 and 84 mV dec^{-1}, respectively, lower than that of CoBDC (98 mV dec^{-1}) and FeBDC (113 mV dec^{-1}). Thus, the universality of this ligand modulation strategy is verified.

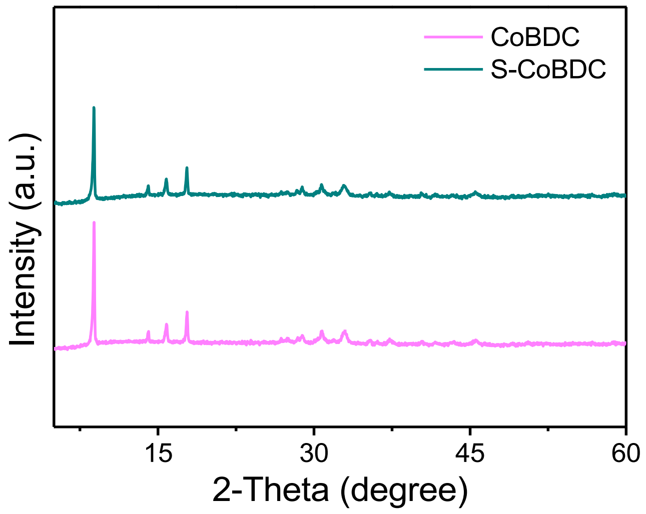

Figure S44. XRD patterns of S-CoBDC and CoBDC.

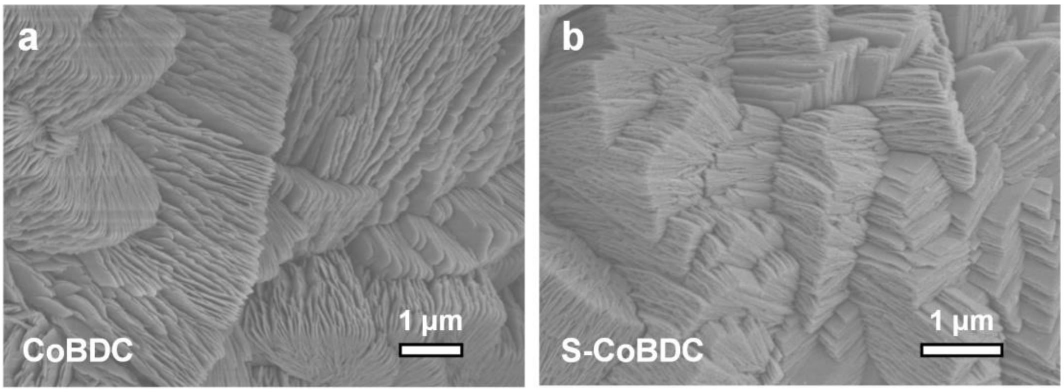

Figure S45. FESEM images of (a) CoBDC and (b) S-CoBDC.

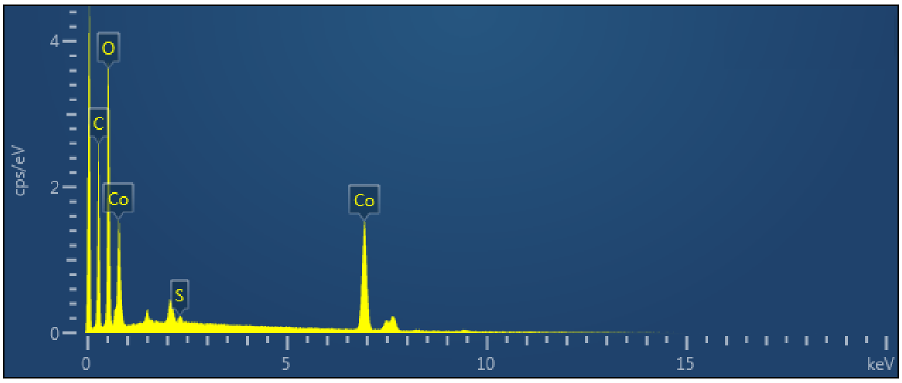

Figure S46. EDX elemental spectrum of S-CoBDC.

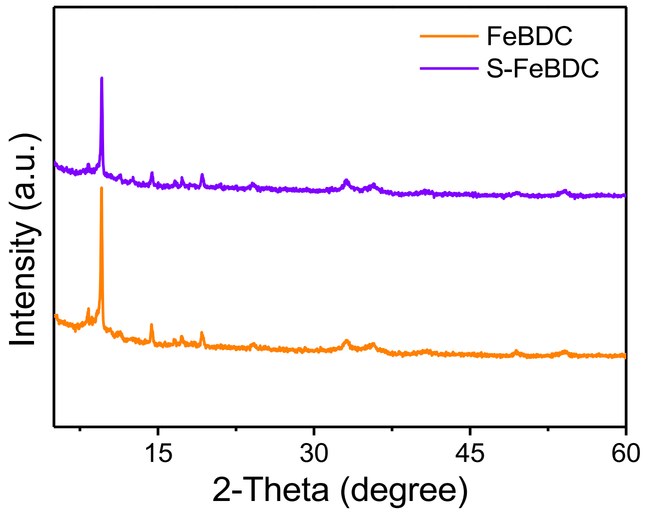

Figure S47. XRD patterns of S-FeBDC and FeBDC.

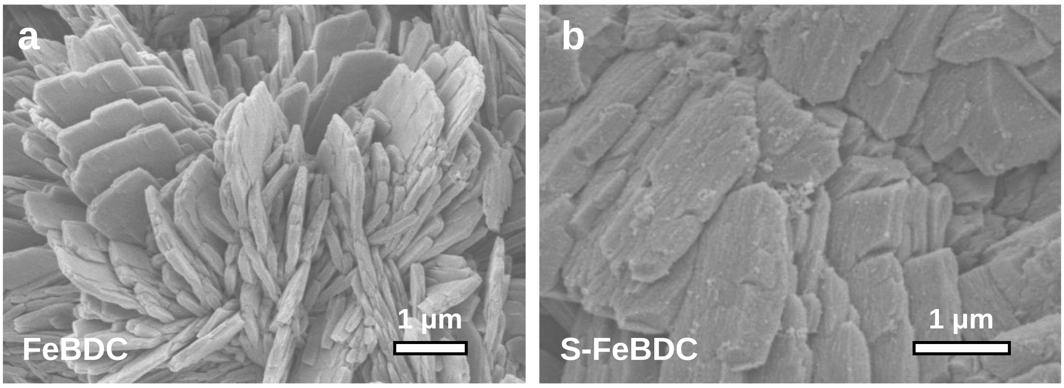

Figure S48. FESEM images of (a) FeBDC and (b) S-FeBDC.

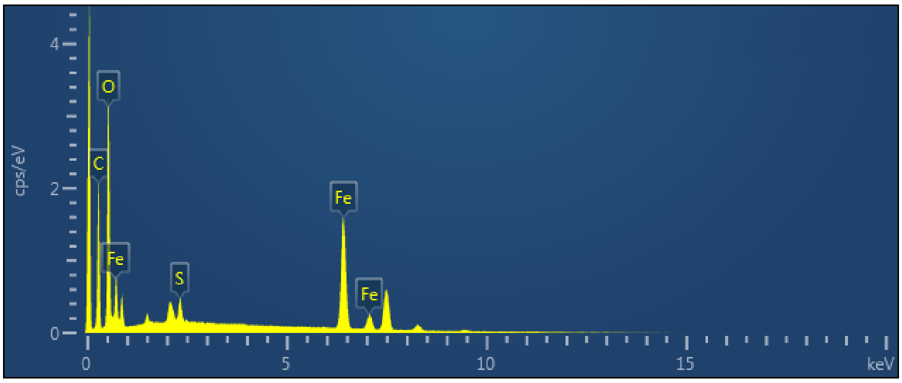

Figure S49. EDX elemental spectrum of S-FeBDC.

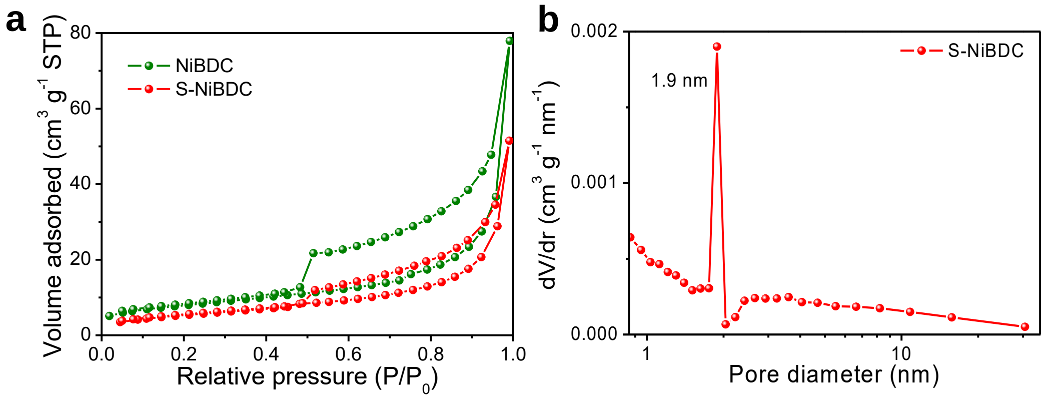

Figure S50. (a) N_2 adsorption-desorption isotherm curves of S-NiBDC and NiBDC. (b) Pore size distribution of S-NiBDC.

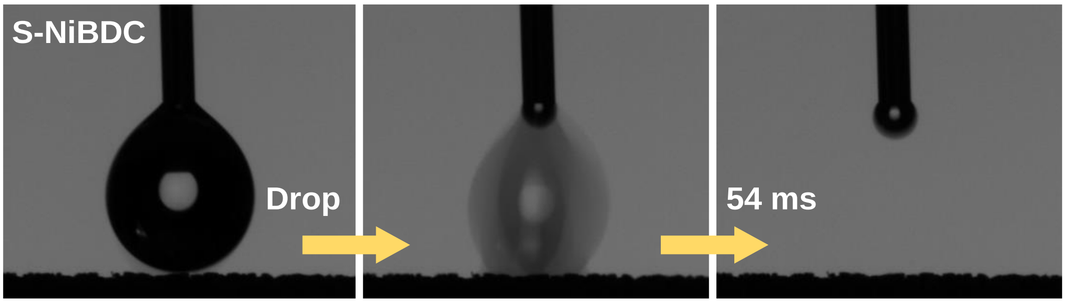

Figure S51. Water-droplet contact angles of S-NiBDC.

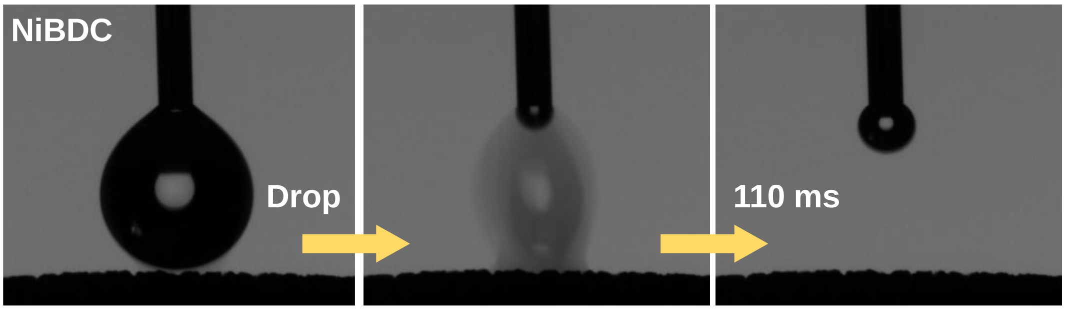

Figure S52. Water-droplet contact angles of NiBDC.

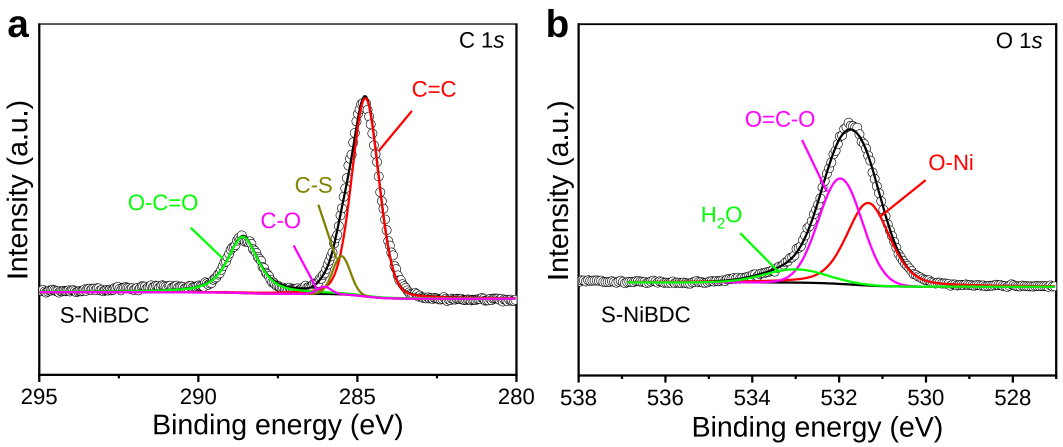

Figure S53. High-resolution XPS spectra for (a) C 1*s* and (b) O 1*s* of S-NiBDC.

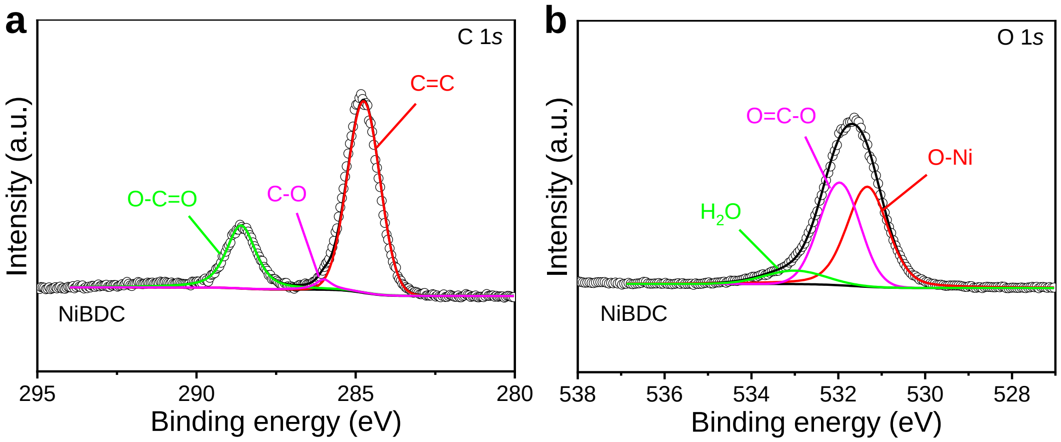

Figure S54. High-resolution XPS spectra for (a) C 1*s* and (b) O 1*s* of NiBDC.

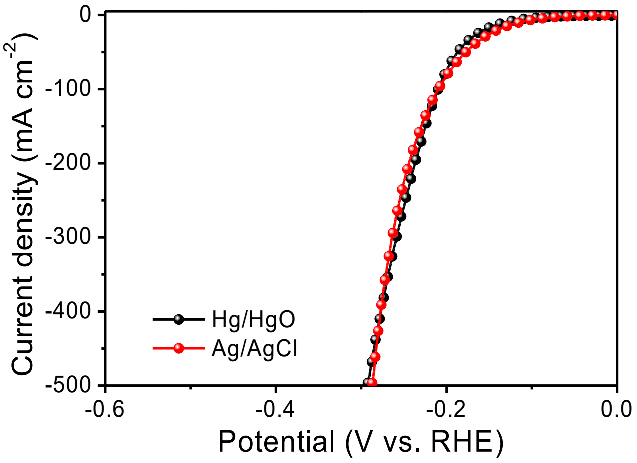

Figure S55. Polarization curves of S-NiBDC using Hg/HgO reference electrode and Ag/AgCl reference electrode.

Owing to the usage of a salt bridge to protect Ag/AgCl reference electrode in this work, the measured HER performance in alkaline media is identical to that measured using Hg/HgO reference electrode.

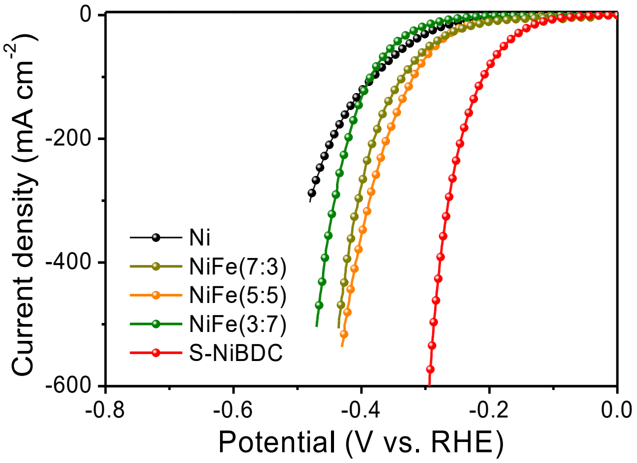

Figure S56. Polarization curves of S-NiBDC and NiFe foam with different metal ratios.

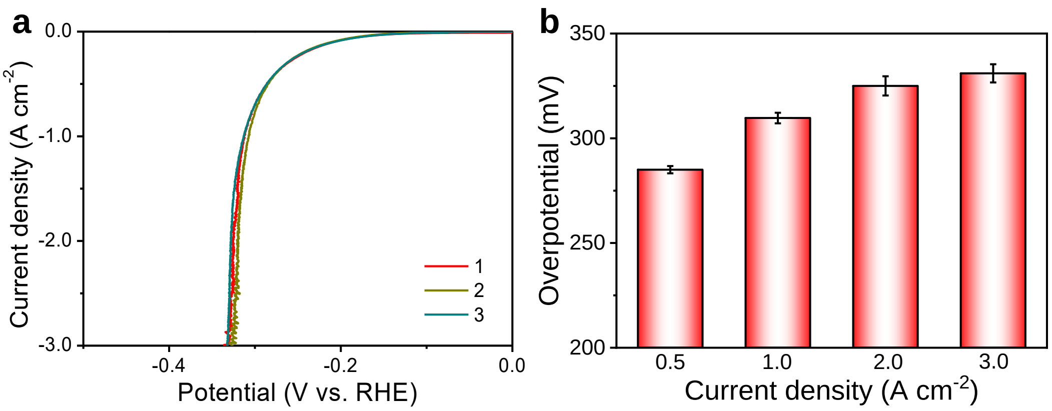

Figure S57. (a) Polarization curves of S-NiBDC prepared in different batches. (b) Overpotentials at 0.5, 1.0, 2.0 and 3.0 A cm^{-2} of S-NiBDC. Error bars correspond to the standard deviation of the three measurements. The reproducibility of samples prepared in different batches were tested for three times.

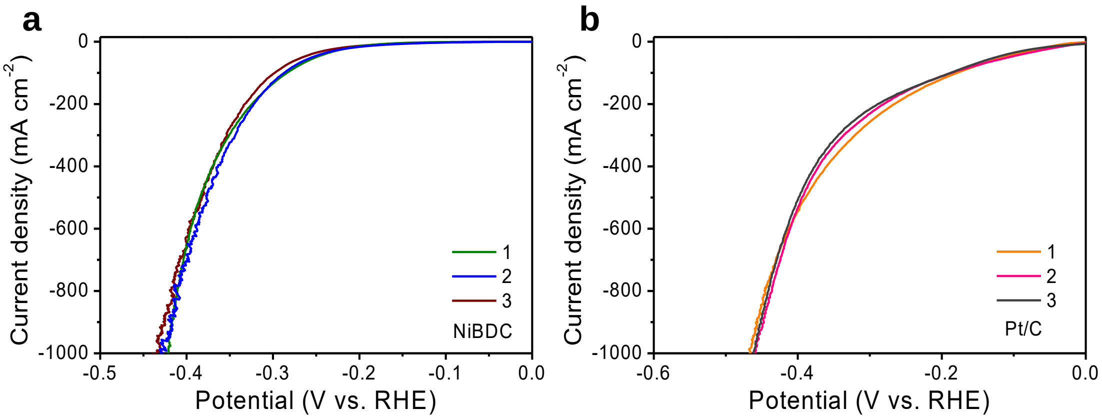

Figure S58. Polarization curves of (a) NiBDC and (b) Pt/C prepared in different batches.

Table S1. EXAFS fitting parameters at the Ni *K*-edge for various samples (S_0^2=0.92).

Sample	Shell	CN^a	R(Å)^b	σ^2(Å^2)^c	ΔE_0(eV)^d	R factor (%)
Ni K-edge						
Ni foil	Ni-Ni	12*	2.47±0.003	0.0047	5.76±0.43	0.3
NiBDC	Ni-O	5.6±0.2	2.03±0.013	0.0091	3.62±1.46	0.7
S-NiBDC	Ni-O	5.1±0.2	1.99±0.022	0.0089	6.41±3.38	0.8
	Ni-S	0.9±0.1	2.15±0.010	0.0076	5.02±2.92	
S-NiBDC-after HER	Ni-O	5.5±0.1	2.08±0.052	0.0096	5.06±5.40	0.3
	Ni-S	0.8±0.3	2.14±0.033	0.0048	4.11±2.37	

^{a}*CN*, coordination number; ^{b}*R*, distance between absorber and backscatter atoms; $^c\sigma^2$, Debye-Waller factor to account for both thermal and structural disorders; $^d\Delta E_0$, inner potential correction; *R* factor indicates the goodness of the fit. S_0^2 was fixed to 0.87, according to the experimental EXAFS fit of Ni foil by fixing CN as the known crystallographic value. Fitting range: 3.0 ≤ *k* (/Å) ≤ 12 and 1.5 ≤ *R* (Å) ≤ 3.0 (Ni foil); 3.0 ≤ *k* (/Å) ≤ 12.0 and 1.0 ≤ *R* (Å) ≤ ~2.2 (Sample NiBDC and S-NiBDC). A reasonable range of EXAFS fitting parameters: 0.700 < S_0^2 < 1.000; *CN* > 0; σ^2 > 0 Å^2; ΔE_0 < 10 eV; *R* factor < 0.02.

Table S2. The EIS results of the catalysts in 1.0 M KOH solution.

Catalysts	R_s/Ω	R_{ct}/Ω
NiBDC	4.33	98.5
S-NiBDC	3.18	19.6

Table S3. Comparison of electrocatalytic HER performances of S-NiBDC with other reported MOF-based HER catalysts in alkaline media.

Catalyst	Electrolyte	Overpotential (mV@mA cm^{-2})	Tafel slope (mV dec^{-1})	Ref.
**S-NiBDC**	**1.0 M KOH**	**113@10**<br>**209@100**<br>**286@500**<br>**310@1.0 A cm^{-2}**<br>**328@2.0 A cm^{-2}**<br>**342@3.5 A cm^{-2}**	**75**	**This work**
NFN-MOF/NF	1.0 M KOH	87@10<br>293@500	35.2	1
Ni-ZIF/Ni-B@NF	1.0 M KOH	67@10<br>282@100	101	2
Co_3S_4/EC-MOF	1.0 M KOH	84@10<br>183@100	82	3
$Ni_3(Ni_3HAHATN)_2$	0.1 M KOH	115@10<br>178@80	45.6	4
Co-MOF@CoP	1.0 M KOH	34@10<br>103@100	56	5
FeNi(BDC)(DMF,F)/NF	1.0 M KOH	160@10<br>259@100	96.2	6
VFe-MOF@NF	1.0 M KOH	147@10<br>406@100	208.25	7
FeMn-MOF/NF	1.0 M KOH	260@50<br>382@100	156.6	8
CoCu-ZIF@GDY	1.0 M KOH	446.5@10<br>588@80	87	9
$Co_{0.4}(Hatz)_{0.1}(H_4bta)_{0.}$	1.0 M KOH	115@10	78	10

1/NF		215@100		
NiFe-MOF-74	1.0 M KOH	195@10<br>591@500	136	11
NiFe-MOF array	1.0 M KOH	68@10<br>356@100	112	12
MFN-MOFs/NF	1.0 M KOH	79@10<br>234@500	30.1	13
NiFe-MOF	0.1 M KOH	134@10<br>288@80	-	14
$Fe(OH)_x$@Cu-MOF	1.0 M KOH	112@10<br>250@60	76	15
Co-BDC/MoS_2	1.0 M KOH	248@10<br>308@40	86	16
MoS_2@CoNi-ZIF	1.0 M KOH	153@10<br>255@50	68	17

Table S4. Comparison of the achieved highest current density and stability time of S-NiBDC with other reported MOF- and metal compound- based HER catalysts in alkaline media.

Catalyst	Electrolyte	Loading amount (mg cm^{-2})	Highest current density (A cm^{-2})	Stability time (h)	Ref.
**S-NiBDC**	**1.0 M KOH**	**1.1**	**3.5**	**150**	**This work**
NFN-MOF/NF	1.0 M KOH	0.6	0.6	30	[1]
Ni-ZIF/Ni-B@NF	1.0 M KOH	-	0.12	64	[2]
Co_3S_4/EC-MOF	1.0 M KOH	3	0.2	24	[3]
$Ni_3(Ni_3HAHATN)_2$	0.1 M KOH	0.566	0.08	5	[4]
Co-MOF@CoP	1.0 M KOH	5	0.2	16.7	[5]
FeNi(BDC)(DMF,F)/ NF	1.0 M KOH	0.5	0.32	30	[6]
VFe-MOF@NF	1.0 M KOH	0.742	0.1	1.11	[7]
FeMn-MOF/NF	1.0 M KOH	-	0.25	12	[8]
CoCu-ZIF@GDY	1.0 M KOH	0.141	0.08	40	[9]
$Co_{0.4}(Hatz)_{0.1}(H_4bta)_{0.1}$/NF	1.0 M KOH	5	0.2	24	[10]
NiFe-MOF-74	1.0 M KOH	-	0.5	15	[11]
NiFe-MOF array	1.0 M KOH	-	0.6	20	[12]
MFN-MOFs/NF	1.0 M KOH	1	0.6	100	[13]
NiFe-MOF	0.1 M KOH	0.3	0.03	0.56	[14]
$Fe(OH)_x$@Cu-MOF	1.0 M KOH	0.1	0.06	30	[15]
Co-BDC/MoS_2	1.0 M KOH	0.159	0.045	15	[16]
MoS_2@CoNi-ZIF	1.0 M KOH	0.143	0.05	48	[17]
Ni-W	1.0 M KOH	0.3	0.675	31	[18]
$Pd_4S/Pd_3P_{0.95}$	1.0 M KOH	0.238	1	20	[19]
MoS_2/Mo_2C	1.0 M KOH	0.3	1.1	24	[20]

Ni_2P/NF	1.0 M KOH	3.83	4.2	24	[21]
Ni_3S_2/Cr_2S_3@NF	1.0 M KOH	1.25	3.5	140	[22]

Table S5. Comparison of electrocatalytic HER performances of S-NiBDC with other reported sulfur-coordinating complex-based HER catalysts.

Catalyst	Electrolyte	Overpotential (mV@mA cm^{-2})	Tafel slope (mV dec^{-1})	Ref.
**S-NiBDC**	**1.0 M KOH**	**113@10**<br>**209@100**<br>**286@500**<br>**310@1.0 A cm^{-2}**<br>**328@2.0 A cm^{-2}**<br>**342@3.5 A cm^{-2}**	**75**	**This work**
CoTHT	pH = 1.3	143@10	70.6	[23]
THTA-Co/G	0.5 M H_2SO_4	230@10	70	[24]
CoBHT<br>NiBHT<br>FeBHT	pH = 1.3	185@10<br>331@10<br>473@10	88<br>67<br>119	[25]
CoBHT<br>CoTHT	pH = 1.3	263@10<br>453@10	149<br>189	[26]
THTNi 2DSP	0.5 M H_2SO_4<br>0.05 M KOH	333@10<br>574@10	80.5<br>-	[27]
Cu-BHT NP	pH = 0.0	450@10	95	[28]
NiAT	0.05 M H_2SO_4	370@10	128	[29]
CoBTT	pH = 1.3	560@10	70	[30]
NiBTT	pH = 1.3	470@10	76	[31]
Co-ATTAc_4/GR	pH = 1.3	388@10	130	[32]
Co-PTC	pH = 0.0	227@10<br>610@100	189	[33]
Cu-MTF	pH = 0.5	96@10<br>463@100	131	[34]

Supplementary References

1 Senthil Raja, D., Chuah, X.-F. & Lu, S.-Y. In Situ Grown Bimetallic MOF-Based Composite as Highly Efficient Bifunctional Electrocatalyst for Overall Water Splitting with Ultrastability at High Current Densities. *Adv. Energy Mater.* **8**, 1801065, (2018).

2 Xu, H. *et al.* Boronization-Induced Ultrathin 2D Nanosheets with Abundant Crystalline-Amorphous Phase Boundary Supported on Nickel Foam toward Efficient Water Splitting. *Adv. Energy Mater.* **10**, 1902714, (2020).

3 Liu, T. *et al.* Self-Sacrificial Template-Directed Vapor-Phase Growth of MOF Assemblies and Surface Vulcanization for Efficient Water Splitting. *Adv. Mater.* **31**, e1806672, (2019).

4 Huang, H. *et al.* Conductive Metal-Organic Frameworks with Extra Metallic Sites as an Efficient Electrocatalyst for the Hydrogen Evolution Reaction. *Adv. Sci.* **7**, 2000012, (2020).

5 Liu, T. *et al.* CoP-Doped MOF-Based Electrocatalyst for pH-Universal Hydrogen Evolution Reaction. *Angew. Chem. Int. Ed.* **58**, 4679-4684, (2019).

6 Lin, H.-W. *et al.* Bi-metallic MOFs possessing hierarchical synergistic effects as high performance electrocatalysts for overall water splitting at high current densities. *Appl. Catal. B* **258**, 118023, (2019).

7 Han, L., Xu, J., Huang, Y., Dong, W. & Jia, X. High-performance electrocatalyst of vanadium-iron bimetal organic framework arrays on nickel foam for overall water splitting. *Chin. Chem. Lett.* **32**, 2263-2268, (2021).

8 Guan, H. *et al.* FeMn bimetallic MOF directly applicable as an efficient electrocatalyst for overall water splitting. *Colloids Surf. A Physicochem. Eng. Asp.* **624**, 126596, (2021).

9 Cui, J. *et al.* Efficient electrocatalytic water oxidation by using the hierarchical 1D/2D structural nanohybrid of CoCu-based zeolitic imidazolate framework nanosheets and graphdiyne nanowires. *Electrochim. Acta* **334**, 135577, (2020).

10 Huang, Z. *et al.* A Facile Reaction Strategy for the Synthesis of MOF-Based Pine-Needle-Like Nanocluster Hierarchical Structure for Efficient Overall Water Splitting. *Inorg. Chem.* **60**, 4047-4057, (2021).

11 Chen, C. *et al.* Tuning the morphology and electron structure of metal-organic framework-74 as bifunctional electrocatalyst for OER and HER using bimetallic collaboration strategy. *J. Alloys Compd.* **865**, 158795, (2021).

12 Sun, Y., Ding, S., Xu, S., Duan, J. & Chen, S. Metallic two-dimensional metal-organic framework arrays for ultrafast water splitting. *J. Power Sources* **494**, 229733, (2021).

13 Senthil Raja, D., Lin, H.-W. & Lu, S.-Y. Synergistically well-mixed MOFs grown on nickel foam as highly efficient durable bifunctional electrocatalysts for overall water splitting at high current densities. *Nano Energy* **57**, 1-13, (2019).

14 Duan, J., Chen, S. & Zhao, C. Ultrathin metal-organic framework array for efficient electrocatalytic water splitting. *Nat. Commun.* **8**, 15341, (2017).

15 Cheng, W., Zhang, H., Luan, D. & Lou, X. W. D. Exposing unsaturated Cu_1-O_2 sites in nanoscale Cu-MOF for efficient electrocatalytic hydrogen evolution. *Sci. Adv.* **7**, eabg2580, (2021).

16 Zhu, D., Liu, J., Zhao, Y., Zheng, Y. & Qiao, S.-Z. Engineering 2D Metal-Organic Framework/MoS_2 Interface for Enhanced Alkaline Hydrogen Evolution. *Small* **15**, 1805511, (2019).

17 Liu, Y. *et al.* Hierarchical nanocomposite electrocatalyst of bimetallic zeolitic imidazolate framework and MoS_2 sheets for non-Pt methanol oxidation and water splitting. *Appl. Catal. B* **258**, 117970, (2019).

18 Wu, H. *et al.* Double-Site Ni-W Nanosheet for Best Alkaline HER Performance at High Current Density >500 mA cm^{-2}. *Adv. Mater. Interfaces* **6**, 1900308, (2019).

19 Zhang, G. *et al.* Interfacial Engineering to Construct Antioxidative $Pd_4S/Pd_3P_{0.95}$ Heterostructure for Robust Hydrogen Production at High Current Density. *Adv. Energy Mater.* **12**, 2103511, (2022).

20 Luo, Y. *et al.* Morphology and surface chemistry engineering toward pH-universal catalysts for hydrogen evolution at high current density. *Nat. Commun.* **10**, 269, (2019).

21 Yu, X. *et al.* “Superaerophobic” Nickel Phosphide Nanoarray Catalyst for Efficient Hydrogen Evolution at Ultrahigh Current Densities. *J. Am. Chem. Soc.* **141**, 7537-7543, (2019).

22 Fu, H. Q. *et al.* Hydrogen Spillover-Bridged Volmer/Tafel Processes Enabling Ampere-Level Current Density Alkaline Hydrogen Evolution Reaction under Low Overpotential. *J. Am. Chem. Soc.* **144**, 6028-6039, (2022).

23 Chen, K., Downes, C. A., Schneider, E., Goodpaster, J. D. & Marinescu, S. C. Improving and Understanding the Hydrogen Evolving Activity of a Cobalt Dithiolene Metal-Organic

Framework. *ACS Appl. Mater. Interfaces* **13**, 16384-16395, (2021).

24 Dong, R. *et al.* Immobilizing Molecular Metal Dithiolene-Diamine Complexes on 2D Metal-Organic Frameworks for Electrocatalytic H_2 Production. *Chem. Eur. J.* **23**, 2255-2260, (2017).

25 Downes, C. A., Clough, A. J., Chen, K., Yoo, J. W. & Marinescu, S. C. Evaluation of the H_2 Evolving Activity of Benzenehexathiolate Coordination Frameworks and the Effect of Film Thickness on H_2 Production. *ACS Appl. Mater. Interfaces* **10**, 1719-1727, (2018).

26 Clough, A. J., Yoo, J. W., Mecklenburg, M. H. & Marinescu, S. C. Two-Dimensional Metal-Organic Surfaces for Efficient Hydrogen Evolution from Water. *J. Am. Chem. Soc.* **137**, 118-121, (2015).

27 Dong, R. *et al.* Large-Area, Free-Standing, Two-Dimensional Supramolecular Polymer Single-Layer Sheets for Highly Efficient Electrocatalytic Hydrogen Evolution. *Angew. Chem. Int. Ed.* **54**, 12058-12063, (2015).

28 Huang, X. *et al.* Conductive Copper Benzenehexathiol Coordination Polymer as a Hydrogen Evolution Catalyst. *ACS Appl. Mater. Interfaces* **9**, 40752-40759, (2017).

29 Sun, X. *et al.* Bis(aminothiolato)nickel nanosheet as a redox switch for conductivity and an electrocatalyst for the hydrogen evolution reaction. *Chem. Sci.* **8**, 8078-8085, (2017).

30 Downes, C. A. & Marinescu, S. C. Efficient Electrochemical and Photoelectrochemical H_2 Production from Water by a Cobalt Dithiolene One-Dimensional Metal-Organic Surface. *J. Am. Chem. Soc.* **137**, 13740-13743, (2015).

31 Downes, C. A. & Marinescu, S. C. One dimensional metal dithiolene (M = Ni, Fe, Zn) coordination polymers for the hydrogen evolution reaction. *Dalton Trans.* **45**, 19311-19321, (2016).

32 Downes, C. A. & Marinescu, S. C. Understanding Variability in the Hydrogen Evolution Activity of a Cobalt Anthracenetetrathiolate Coordination Polymer. *ACS Catal.* **7**, 8605-8612, (2017).

33 Chen, Z. *et al.* Highly Conductive Cobalt Perthiolated Coronene Complex for Efficient Hydrogen Evolution. *Chem. Eur. J.* **26**, 12868-12873, (2020).

34 Chatterjee, S. *et al.* Metal-Thiolate Framework for Electrochemical and Photoelectrochemical Hydrogen Generation. *ChemSusChem* **15**, e202200114, (2022).
